# Development of Flotation Device for Removing Unburnt Carbon in Fly Ash for Use in Hardened Cementitious Materials

**DOI:** 10.3390/ma14216517

**Published:** 2021-10-29

**Authors:** Hangwei Lin, Koji Takasu, Hidehiro Koyamada, Hiroki Suyama

**Affiliations:** Department of Architecture, Faculty of Environmental Engineering, The University of Kitakyushu, Kitakyushu 808-0135, Japan; a9dbb419@eng.kitakyu-u.ac.jp (H.L.); h-koyamada@kitakyu-u.ac.jp (H.K.); suyama@kitakyu-u.ac.jp (H.S.)

**Keywords:** flotation, fly ash, concrete, unburnt carbon

## Abstract

The unburned carbon in fly ash inhibits the performance of concrete. A device using the flotation method to remove unburned carbon in fly ash was developed, and the operating condition of the device was experimentally examined. According to the results, the device was able to remove unburnt carbon from fly ash by using the installed micro bubble nozzles and a whirl-type pump. The removal efficiency of unburnt carbon improved when prior forced stirring was carried out by a concrete mixer for 3 min, and a scavenger was added into the fly ash slurry at a density of about 60 wt%. It has also been confirmed that the method of circulating water is more effective than the method of not circulating water. The elements of the modified fly ash slurry (MFAS) have also been experimentally confirmed as not being too different from untreated fly ash, except for the fact that the content of unburned carbon is reduced. The compressive strength and drying shrinkage characteristics of concrete made with MFAS were investigated. The use of MFAS will reduce the performance of concrete compared to that of ordinary concrete. This shows that in a certain range (15–30%), the influence of MFAS on drying shrinkage is constant. The static elastic modulus and dynamic elastic modulus were also investigated. The above results show that the application of MFAS prepared by the flotation method to concrete is feasible.

## 1. Introduction

In Japan, the power supply includes renewable [1], thermal [2], and nuclear energy [3]; however, global environmental issues and the recent power supply situation mean that renewable energy is expected to expand substantially. Nevertheless, increasing the proportion of renewable energy in the energy mix will take time, and thermal power generation will still be required.

Coal-fired power generation has the second-highest utilization rate worldwide after liquefied natural gas power generation, but the treatment of the coal ash generated is a major problem. Fly ash is the fine residue generated by the combustion of ground or powdered coal and is transported through flue gasses. Global fly ash production is estimated to be 400–500 million tons per year and the utilization rate in cement and concrete components is about 30% [4,5]. The total amount of coal ash generated by Japan’s electric power industry and general industry exceeded 10 million tons and 97.4% of the coal ash was used, and 96.3% of the total was used in the cement industry as a raw material.

The properties of recycled aggregate concrete (RAC) cause major problems globally and improving the properties of RAC is expected to increase the use of RAC in structures. Recycled aggregates generally increase the water absorption and drying shrinkage and reduce the modulus of elasticity, workability and compressive strength of RAC compared with concrete containing natural aggregate [6]. However, fly ash has a shrinkage-reducing effect on both ordinary concrete and RAC and can mitigate the increase in shrinkage caused by recycled aggregates [7]. For concrete with a water:cement ratio of 0.55, the dry shrinkage of concrete prepared with 0%, 20%, 50%, and 100% recycled aggregate is reduced by 14%, 13%, 10%, and 7%, respectively [8]. When 35 wt% of cement is replaced with fly ash, the shrinkage strain of all types of concrete is decreased by 55 × 10^−6^ on average, and the shrinkage strain of concrete without fly ash is 15–20% higher than that with fly ash at an age of 112 days [9]. Using fly ash as an alternative to cement or as an additive that can improve the durability and workability of the concrete is the recommended approach and helps to protect the environment and reduce water consumption. The strength of concrete in which a percentage of cement is replaced by fly ash is lower at an early age, although its strength is higher than or similar to concrete without fly ash later on [10,11,12]. In general, concrete containing fly ash as an additive or to replace cement has improved durability. The higher compressive strength of concretes containing fly ash is related to the improved bonding between aggregates and slurry and the denser microstructure obtained by changing the pore size distribution [13]. 

Fly ash is classified into types I to IV according to Japanese Industrial Standard (JIS), and the quality of fly ash must be considered when it is used as an admixture for concrete. According to the Architectural Institute of Japan, type I or JASS5M-401 [14] fly ash is suitable for replacing cement, and types II or IV are suitable for replacing a proportion of fine aggregate. In other words, fly ash used as an admixture for concrete requires a loss on ignition (LOI) of 5.0% or less. This is because fly ash with an unburned carbon content of 5.0% or more may cause poor coagulation of cement, and that with an unburned carbon content of 3.0% or more, adsorbs admixtures and decreases the fluidity and workability of concrete, which prevents entrainment and adversely affects the concrete’s quality [15].

Some fly ash discharged from coal-fired power plants has a LOI of more than 5.0%, but other qualities mainly conform to JIS standard. Even for fly ash with a LOI of less than 5.0%, the smaller the amount of unburned carbon, the smaller the effect on the fresh properties of concrete. Therefore, methods of separating unburned carbon from fly ash will help increase the use of fly ash as an admixture for concrete and the reuse of the fine unburned carbon, achieving more efficient use of waste fly ash and greater economic and environmental benefits compared with landfill disposal or storage. Methods for separating unburned carbon particles from fly ash and the effect of the method on the unburned carbon properties have been investigated.

In general, high-carbon fly ash can be beneficiated using dry and wet separation processes, such as froth flotation, electrostatic separation, fluidized bed reactors, oil agglomeration, density separation, and sieving [16,17,18,19]. Each separation method has disadvantages and advantages and can be adapted for different types of coal fly ash. For improved separation efficiency and higher purities of unburned carbon, a combination of several techniques is typically needed. For example, Bittner et al. [20] developed a processing system based on triboelectric charging and electrostatic separation, and Parallel and louvered plate separators were used for the beneficiation of fine coal fly ash particles by Soong et al. [21].

The flotation method is a conventional technique that is mainly used for coal beneficiation, ore beneficiation, and the deinking of used paper [22,23]. Hydrophobic particles are attached to the surface of bubbles and floated in the water, allowing for hydrophilic particles to be collected from the bottom. To improve the separation, a foaming agent and a collecting agent that improves particle adhesion to bubbles are often added [24,25]. Froth flotation is widely used in mineral processing and coal preparation industry [26]. The traditional Denver flotation cell has been used for the separation of unburned carbon in laboratory scale fly ash [16]. The existence of a large number of pores increases the consumption of diesel. During the adjustment process, the amount of diesel and the speed of the impeller have the greatest impact on the carbon recovery rate. Altun et al. [27] used a concurrent flotation column. Unburned carbon was separated from fly ash, and the effects of gas flow, pH value, the amount of collected kerosene and different types of fly ash on the separation performance were investigated. It was concluded that column flotation was an effective method. Li et al. [28] developed a flotation method, which is a novel device with the characteristics of an internal recycling process and multiple mineralization steps. Uçurum et al. [29] found that in trials of a flotation method, unburned carbon was removed from the fly ash, but they did not determine the effectiveness of the removal effect. With the technique disclosed in past research, since separation cannot be performed efficiently, treatment requires a long time period, thus preventing sufficient productivity from being obtained and the floatation machine then becomes complicated and large, requiring extremely large installation space and a high facility cost. It is therefore impossible for small to medium fresh concrete factories to install such facilities.

In floating ore and coal beneficiation equipment, air bubbles are discharged from the bottom of a cylindrical container by an air diffuser and push the carbon over the top of the container. To use this equipment to remove unburned carbon from fly ash, the following factors must be studied: the effect of the bubble size; the methods for recovering fly ash from which the unburned carbon has been removed and for recovering the fly ash containing a large amount of unburned carbon; the method of recovering the fly ash from the fly ash slurry; the stirring method; the operating conditions of the device; and the types of device that can remove unburned carbon efficiently.

In this research, we prototyped an unburned carbon removal device for separating unburned carbon from fly ash by the flotation method. The first objective is to provide a floatation separation apparatus in a simple structure capable of efficiently separating materials to be treated, and the second objective is to provide a simple floatation separation method for efficiently separating unburnt carbon contained in fly ash. The third objective is to provide a simple manufacturing method for efficiently manufacturing a cement mixture using high-quality fly ash with a reduced unburnt carbon content. We investigated the size of bubbles suitable for removal and the operating conditions. We measured the effect of the conditions on the carbon removal and optimized the device design. Since the unburnt carbon content of the fly ash slurry thus obtained was decreased sufficiently, unburnt carbon-related problems hardly occur, even if a large amount is used, and whether such fly ash slurry can be used as various raw materials in large amounts is worthy of investigation. Therefore, the mechanical and physical properties of concrete containing 15% or 30% fly ash and modified fly ash slurry (MFAS) with unburned carbon removed with our device were measured.

## 2. Materials and Methods

### 2.1. Flotation Method Experiment

In the initial experiment, we examined the effects of the bubble diameter and the conditions on the unburned carbon removal to develop a base model of an unburned coal removal device, and the experiments described later were carried out step-by-step from devices I to III.

Table 1 shows the physical characteristics of the fly ash, which was generated from two thermal power plants. Fly ash a-2, a-3, a-5, and a-6 corresponded to JIS A 6201 [30] type II; a-4, b-1, b-2, and b-3 corresponded to type III; a-1 corresponded to type IV; and b-4 did not correspond to a defined type due to its high LOI.

**Figure 1:**
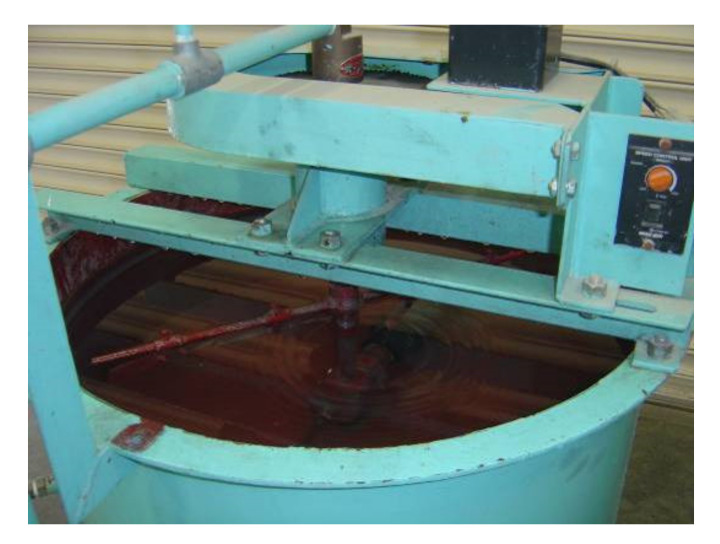
The flotation device of the air diffuser.

Experiment I was performed to examine the effect of bubble diameter. In device I, which had a capacity of 80 L, fly ashes from the ‘a’ series were used, and two general-purpose air diffusers Ø 70 mm × L200 mm in size were attached to the flotation device near the bottom (Figure 1 is based on the equipment used for coal flotation). The air was agitated at a rotation speed of 19 rpm, and the amount of air discharged from the air diffuser was 165 L/min. The most frequent bubble diameter was about 200 μm. The foaming agent was 0.3% pine oil and the unburned carbon collector was 5.0% kerosene. The device floated fly ash containing a large amount of unburned carbon near the water surface through aeration with the air diffuser, and then the floating material (foam ash) was removed and the fly ash that settled on the bottom after standing (tail ash) was collected. The experiment was performed as follows. The additives and tap water were inserted into the device and stirred for 5 min, fly ash was added to make a 20 wt% fly ash slurry and was stirred for 5 min, and then the mixture was aerated for 10 min before the foam ash was recovered. The tail ash was collected from the bottom of the device and dried in an electric furnace at 105 °C for 24 h and its LOI was measured. The unburned carbon removal rates of the ash types were determined based on the LOI measured by JIS A6201 [30].

**Figure 2:**
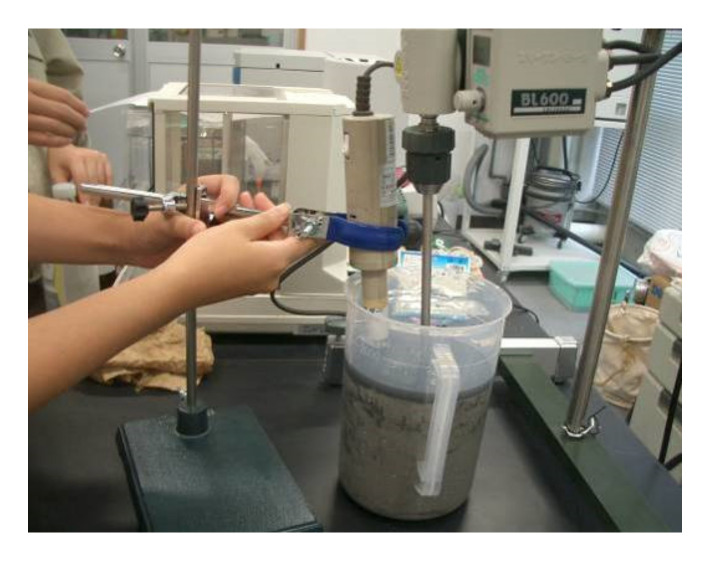
Microbubble generator.

Experiment II examined the effect of the bubble diameter and used fly ashes from the ‘b’ series, which had a larger LOI than fly ashes from the ‘a’ series. In device II, the fly ash was put into a 2 L plastic container (Figure 2). To prevent precipitation, a small mixer with a rotation speed of 250 rpm was used for mixing, and a high-speed rotary blade microbubble generator was used. The mode of the microbubble diameter was about 40 μm. In addition, in device II, a 60 wt% fly ash slurry and the collector were pre-stirred in a 0.6 L high-speed mixer before flotation due to the significant improvement in the separation performance of unburned carbon from coal fly ash [31]. Pretreatment has great importance for the flotation method. The kinetic energy that is dissipated in the stirred tank could strengthen the interaction process between mineral particles and flotation reagents to improve the flotation efficiency in the presence of preconditioning [32]. Yu et al. [33] found in the flotation of coal that high intensity agitation, greater than 1200 rpm, reduces the kaolinite coating, which will lead to a higher combustibles recovery rate. The collecting agent and tap water were placed in the mixer (rotation speed 10,000 rpm), stirred for 1 min, the foaming agent was added, and the slurry concentration was increased to 20 wt%. Then, the air rotation was started and the floss ash was collected for 10 min. The additive addition rates were the same as those for experiment I. The device was left to stand, the tail ash was collected from the bottom of the device and dried, and then the LOI was measured in the same way as in experiment I.

**Figure 3:**
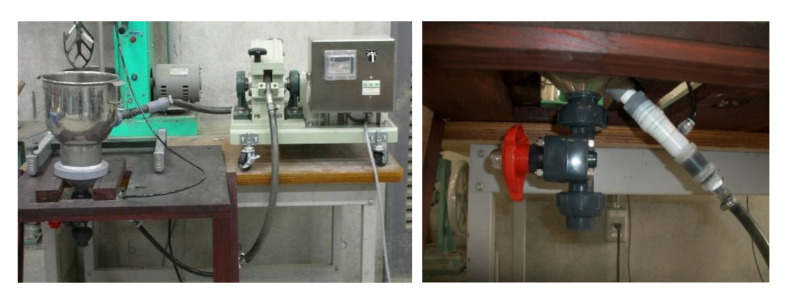
Microbubble circulation device.

Experiment III was conducted to collect data for designing the actual device used in the industry and confirming the effectiveness of preprocessing based on the results of device II. The same fly ash as for device II was used as the material. Device III consisted of a 5 L metal container (Figure 3) and the fly ash slurry was circulated by a roller pump with a built-in orifice microbubble generator to prevent the ash from settling without using a stirrer. The experimental procedure was the same as that of experiment II, but the results with and without pre-stirring were compared.

### 2.2. Concrete Specimen Fabrication

Concrete specimens were fabricated using MFAS and type II fly ash. Ordinary Portland cement was used, with sea sand as a natural fine aggregate, and crushed stone was used as a coarse aggregate. The properties of raw ash and MFA were tested (Table 2). Fly ash was used to partially replace cement. The JIS R 5210 [34] and JIS A 6201 [30] standards were followed.

The mixture proportions are shown in Table 3. The fine aggregate, cement, and fly ash were mixed for 30 s, water was added and mixed for 60 s, and coarse aggregate was added and mixed for 60 s.

### 2.3. Concrete Specimen Tests

The compressive strength test was conducted using cylindrical specimens (Ø = 100 mm, h = 200 mm) according to JIS A 1108 [35]. For each mix, 12 cylinders were cast in a mold and kept in a chamber at 20 °C and 60% relative humidity for 24 h, after which they were demolded. The ages of the tested specimens were 1, 4, and 13 weeks. In addition, a specimen was tested immediately after the curing was complete. The load was applied at a uniform rate to avoid subjecting the specimen to impact loading; the loading rate was such that the compressive stress increased by 0.6 ± 0.4 N/mm^2^ per second. During each test, the static elastic modulus specimens stored at the temperature and humidity specified for the test were tested (JIS A 1149 [36]). 

The drying shrinkage test was conducted using a cuboid (100 × 100 × 400 mm^3^) according to JIS A 1129-3 [37]. For each mix, three cuboids were cast in a steel mold and kept in a chamber at 20 °C and 60% relative humidity for 24 h until demolded. After demolding, the concrete specimens were immersed in water at 20 ± 2 °C and cured for 7 days. During the drying period, the specimens were kept at least 25 mm apart so as not to impede drying from the bottom of the specimen. Measurements were taken when a specimen was 7 days old, and this time was taken as the reference.

Dynamic elastic modulus tests were conducted using a cylinder or cuboid placed on a support base so that both ends could vibrate freely without being restrained. The output voltage of the amplified pickup was observed, and the frequency at which the indicator had a clear maximum vibration was defined as the primary resonance frequency of the longitudinal vibration according to JIS A 1127 [38].

## 3. Results and Discussion

### 3.1. Removal of Unburned Carbon by the Flotation Method

**Figure 4:**
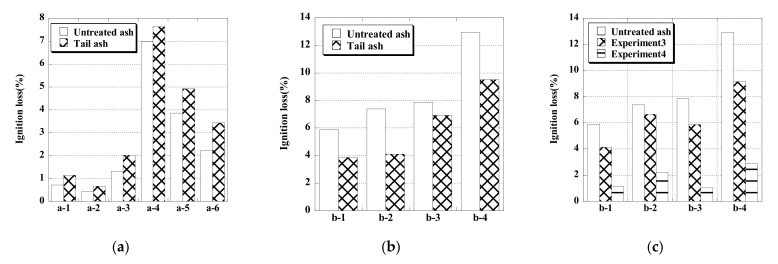
LOI of untreated ash and tail ash in experiment I (**a**), experiment II (**b**) and experiment III (**c**).

Figure 4 shows the LOI of untreated ash and tail ash for each device. When a diffuser tube was used in experiment I (Figure 4a), the LOI of the tail ash for all fly ash was slightly larger than that of the untreated ash in the range of this experiment, showing that the removal method had no effect. However, in experiment II (Figure 4b), the microbubble generator reduced the LOI and removed unburned carbon from the fly ash. In experiments I and II, it was not possible to compare samples from the same discharge source, but the decrease in LOI was due to the difference in equipment rather than the fly ash characteristics. One reason for the larger LOI in experiment I was that the mode of the bubble diameter generated from the air diffuser was about 200 μm, which was about 10 times larger than that of the fly ash particles, and thus it was difficult to collect or raise the unburned carbon to the surface. In contrast, in experiment II, the mode of the microbubble diameter was 40 μm, which was about twice as large as that of the fly ash particles, allowing the unburned carbon to be efficiently collected and raised to the surface. Therefore, microbubbles were effective in removing unburned carbon in fly ash by the flotation method.

Next, we compare the results of experiment II, in which pre-stirring was performed, with those of experiment III. For fly ashes b-1 to b-4, the LOI was lower in experiment III than in experiment II. In particular, in experiment II, the LOI was reduced to 3.0% or less by using the circulating microbubble generator in device II, and the fly ash was modified to the equivalent of JIS type I. The circulating microbubble generator prevented the fly ash from settling without a stirrer, and the microbubbles were in uniform contact with the fly ash particles, and so the microbubbles enclosed the unburned carbon collected by the collector, which increased the effectiveness of the device. The effect of pre-stirring in the circulating microbubble generator was examined in experiment III. In the absence of pre-stirring, the LOIs of the tail ash of all types of fly ash were slightly lower than those of the untreated ash (Figure 4c). In contrast, when pre-stirring was performed, the LOIs of the tail ash were 3.0% or less for all types of fly ash, and the LOI could be decreased by up to 82%. Especially for fly ash b-4, the untreated ash did not correspond to a JIS type, but the tail ash had an LOI equivalent to JIS type I.

**Figure 5:**
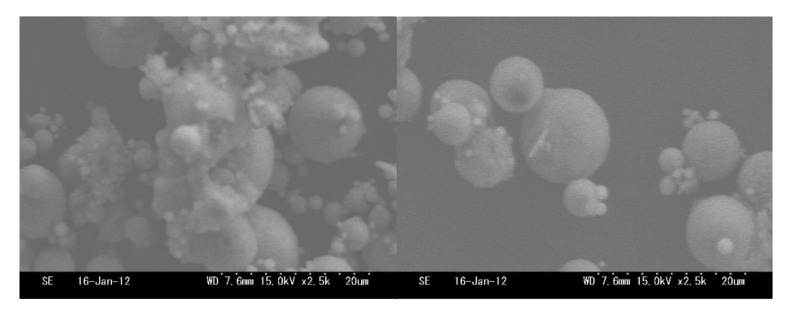
SEM images of raw ash and tail ash of b ash-1.

Figure 5 shows scanning electron microscope images of untreated ash and tail ash of b-1. Unburned carbon and other deposits were attached to the untreated fly ash particles, whereas the unburned carbon was physically removed from the surface of the tail ash particles. Pre-stirring most likely removed the unburned carbon from the fly ash particles and explained why pre-stirring decreased LOI substantially. Therefore, these results showed that for the flotation method using a microbubble generator, unburned carbon in the fly ash was effectively removed by pre-stirring using a mixer. The LOI of the foam ash was in the range of 55 to 70 wt%, indicating that it contained a large amount of unburned carbon and could be used as an auxiliary fuel after drying.

### 3.2. Base Model Development and Performance Verification

**Figure 6:**
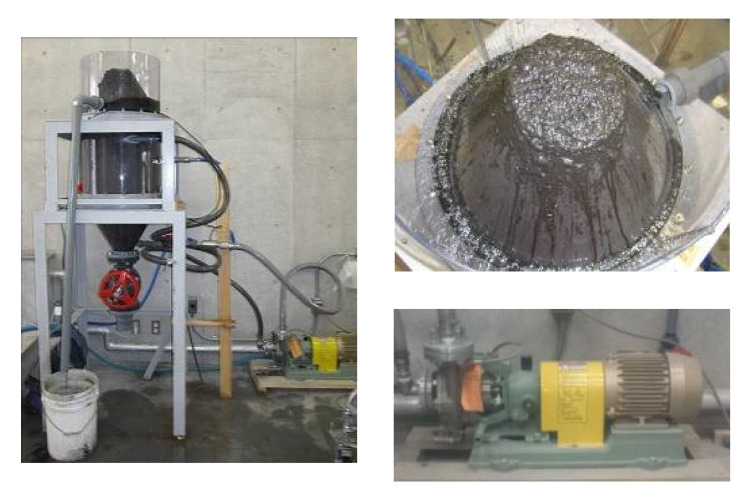
The medium unburned carbon removal device.

**Figure 7:**
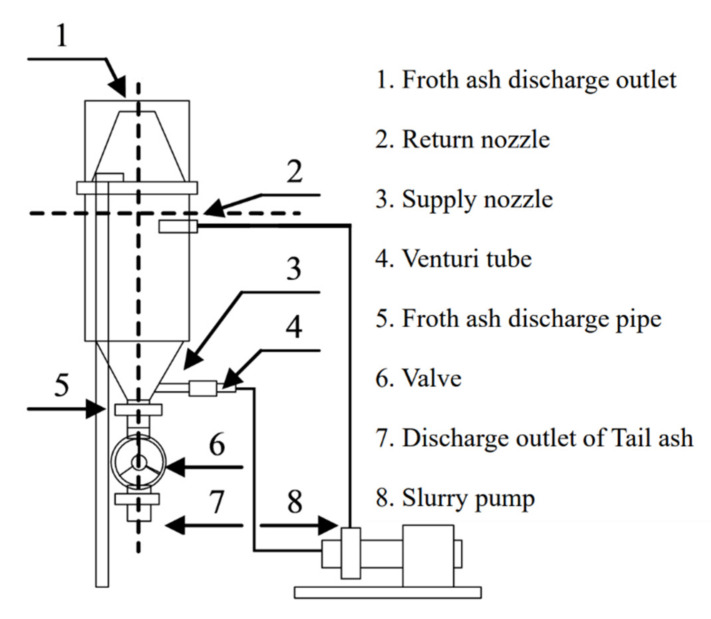
The draw of medium unburned carbon removal device.

Based on the results of experiments I–III, we developed a base model of an unburned coal removal device that used the flotation method. Figure 6 shows the base model of the device, the spiral circulation pump, and the removal of foam ash. Figure 7 shows the draw of the base model of the device. The capacity of the device was 130 L. In experimental devices I–III, a roller pump was used to circulate the fly ash slurry, but in the base model, a spiral circulation pump with a higher circulation capacity was used. This type of pump can be used in an actual plant with a capacity of 10 m^3^ or more. In the base model, the microbubble generator was placed eccentrically at the bottom of the side surface of the device to generate a vortex, and the top of the device was conical. This vortex attracts the foam ash to the center and causes the foam ash to flow from the upper part of the device so that it can be automatically discharged. The microbubble diameter was 40 μm, the pump output was 0.4 kW, the pump flow rate was 30 L/min, and the maximum air supply was 10 L/min. Compared with the traditional flotation technology, it does not need mechanical agitation and has the advantages of a more compact design and lower capital cost.

**Figure 8:**
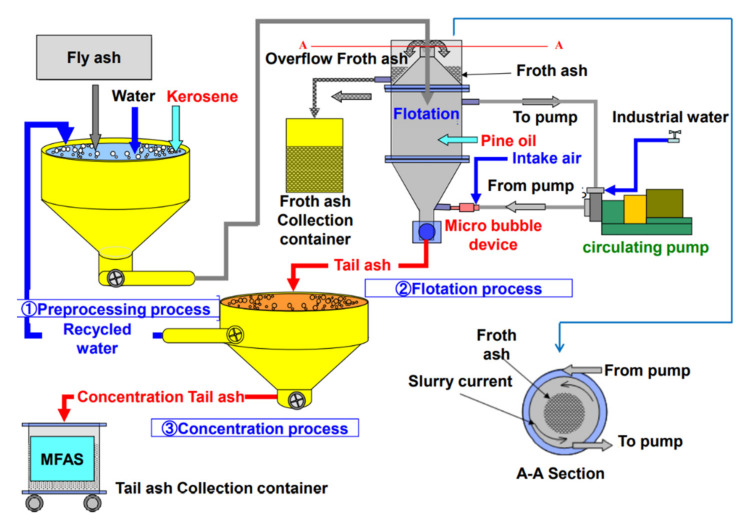
The flotation process of using medium unburned carbon removal device.

Figure 8 shows the process of the flotation method using this device. The device is composed of the flotation tank, the circulating pump, and the microbubble generator. Fly ash, kerosene, and water are mixed as a prior process, and it is supplied to the flotation tank. The circulating pump is operated, and microbubbles are blown in from the lower side of the flotation tank. The froth ash that contains most of the unburned carbon accumulates and is expelled from the upper part of the flotation tank. The tail ash from which unburned carbon is removed accumulates below and is expelled from the lower side of the flotation tank.

Fly ashes c and d generated from the two thermal power plants from Japan were used (Table 1). Fly ash c was equivalent to JIS type III, and fly ash d did not conform to JIS standards. The experimental procedure was the same as for experiment III with pre-stirring, but because the device capacity was 130 L and the number of input samples was large, pre-mixing was performed with a 50 L concrete mixer (MARUI & CO., LTD, Daito, Japan) (speed 50 rpm) to eliminate the effect of the rotation speed caused by the insufficient capacity of the 0.6 L high-speed mixer. However, the rotation speed of the pre-stirring was 1/200th of that in experiment III. To make the total rotation speed of the mixer approximately the same as that in experiment III, the flotation beneficiation time was set to 30 min to consider the increased capacity of the device. The additive addition rate was the same as in experiment III.

Table 4 shows the physical characteristics of untreated ash and tail ash for fly ashes c and d. In all ashes, the LOI of the tail ash decreased to 3.0% or less compared with the untreated ash, the specific surface area decreased, and the density increased due to the removal of unburned carbon, which is porous and amorphous. Fly ash that is usable as various materials, preferably the fly ash with an unburnt carbon content as low as 5 wt%, and more preferably the fly ash with an unburnt carbon content as low as 3 wt%, can be obtained efficiently with a simple structure.

**Figure 9:**
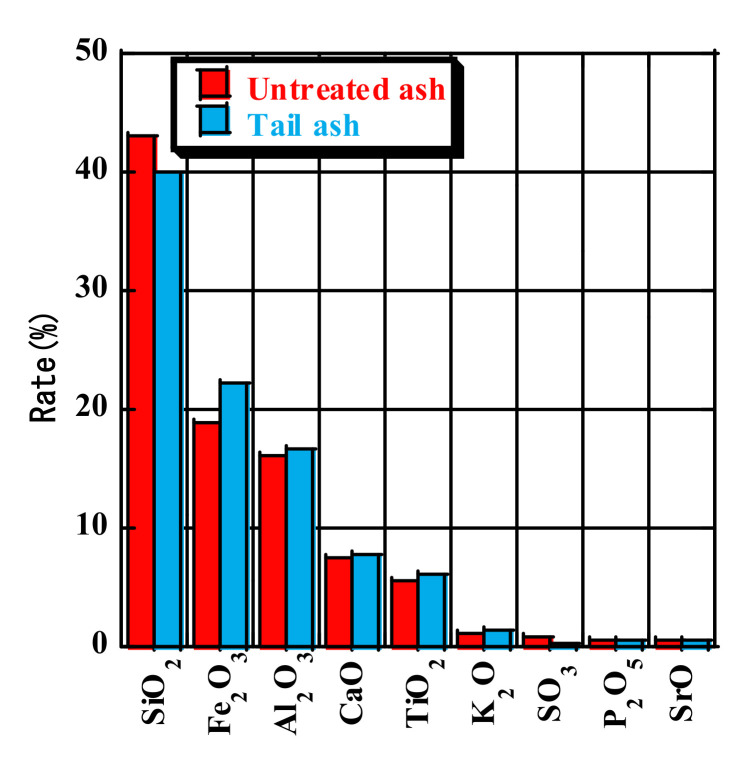
Chemical components of untreated ash and tail ash.

Figure 9 shows the chemical composition of untreated ash and tail ash obtained by fluorescence X-ray analysis. The chemical composition was analyzed to measure the chemical effects of removing unburned carbon. Some components of the tail ash increased slightly when the LOI decreased, although none of the components changed substantially. Therefore, the unburned carbon removal device did not affect the chemical composition of the fly ash, other than removing the unburned carbon. 

### 3.3. Treatment Conditions

Pre-stirring enhances the interaction between the collector and the fly ash. However, too much energy input from the stirring tank cannot improve the flotation efficiency. Flotation process factors of pre-stirring greatly affect the flotation capacity and efficiency. We examined the effect of the pre-stirring time, fly ash slurry concentration during flotation treatment, flotation processing time, and chemicals, and the optimum treatment conditions, such as the addition rate, in device III. Fly ash e was used in the tests (Table 1). Ash e was equivalent to JIS type II, but because the conditions strongly affect the removal of unburned carbon, untreated ash with a low LOI was selected. First, the pre-stirring time and flotation processing time were examined. The concentration of the fly-ash slurry was set to 60%, and the slurry was pre-stirred using a concrete pan mixer (rotation speed 50 rpm). Then, the slurry concentration was adjusted to 6.6 wt%, and flotation was performed. The chemical addition rate was the same as for experiments I–III (collecting agent 5.0% and foaming agent 0.3% with respect to the fly ash mass). After determining the appropriate treatment time, the slurry concentration during the flotation treatment was changed to examine the treatment efficiency, and finally the chemical addition rate was changed. The microbubble diameter was 40 μm, the pump output was 0.4 kW, the pump flow rate was 30 L/min, and the maximum air supply was 10 L/min.

**Figure 10:**
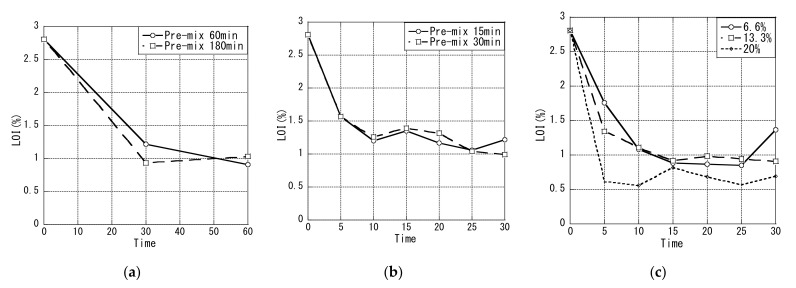
LOI of the flotation treatment with 60 or 180 min (**a**) and 15 or 30 min (**b**) pre-stirring, and at slurry concentrations of 6.6, 13.3, and 20.0 wt% (**c**).

Figure 10a shows the LOI after 60 min of flotation treatment with 60 or 180 min of pre-stirring. The LOI was measured during the flotation treatment by sampling the tail ash from the tail ash outlet at 30 and 60 min without stopping the equipment. At 60 min, LOI was 0.12% lower for a pre-stirring time of 180 min; thus, the effect of the pre-stirring time on the reduction inLOI was small. After 30 min, there was a large reduction in LOI, and a smaller change after 60 min, which suggested that the flotation treatment time could be shortened to 30 min or less.

Figure 10b shows the time course of the tail ash LOI sampled every 5 min up to a flotation time of 30 min with a pre-stirring time of 15 or 30 min. LOI was greatly reduced within 10 min. Because decreasing the pre-stirring time from 60 min to 15 min reduced the LOI, and there was almost no difference in LOI between pre-stirring times of 15 and 30 min, in the subsequent experiments the pre-stirring time and flotation treatment time were each set to 30 min for safety considerations.

In the flotation treatment, the efficiency of one treatment increased when the fly ash slurry concentration was increased. Therefore, we investigated the change over time in LOI at slurry concentrations of 6.6, 13.3, and 20.0 wt% (Figure 10c). At all fly ash slurry concentrations, the reduction in LOI was large within 10 min, and the smallest reduction was for a fly ash slurry concentration of 20.0 wt%. A preliminary experiment with a slurry concentration of 25.0 wt% showed that the reduction rate of LOI was lower than that at 20.0 wt% (data not shown). Therefore, the floating beneficiation method was efficient at a slurry concentration of 20.0 wt%.

**Figure 11:**
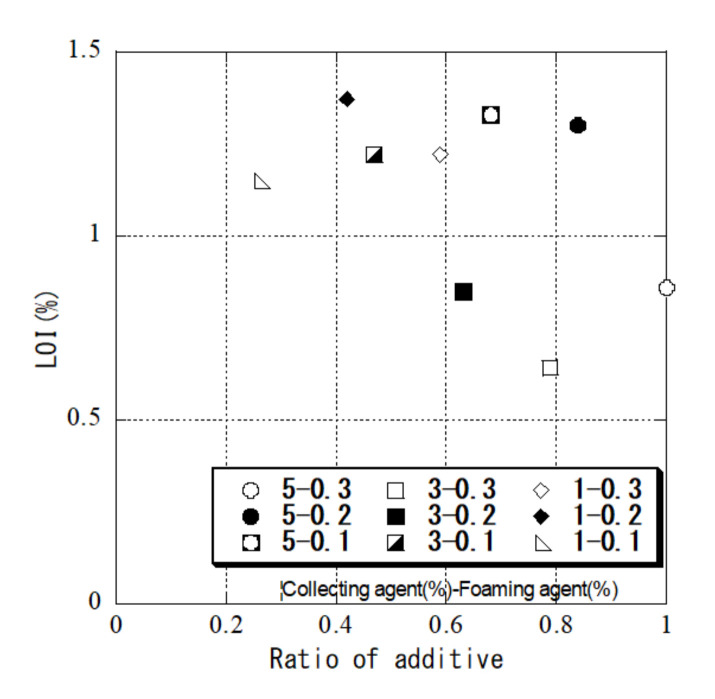
Relationship between the agent ratio and LOI under each condition.

The addition rates of the collecting agent and foaming agent were examined because they affect the processing cost. Flotation treatment was performed under a total of nine conditions at different mixing ratios. Here, we set the fly ash slurry concentration to 6.6 wt%. Figure 11 shows the relationship between the additive cost ratio and LOI under each condition, where the additive cost for treatment with 5.0% collecting agent and 0.3% foaming agent is 1.0. The additive cost is the unit price when 18 L of the collecting agent and 18 L of the foaming agent are purchased as laboratory chemicals, and the LOI was measured after 30 min treatment by the flotation method. Assuming that the LOI control value was 1.0% or less, the best mixing ratio was 3.0% for the collecting agent and 0.2% for the foaming agent, which was a reduction of about 40% for the collector and about 33% for the foaming agent compared with the initial conditions. However, because the additive addition rate depends on the control value of LOI, the rate must be set at the operation stage, and the optimum mixing ratio can be determined by conducting the same experiment. In addition, the results show that the removal of unburned carbon was high for a ratio of 3.0% for the collecting agent and 0.3% for the foaming agent. The unburned carbon removal did not increase with the collection agent addition rate, whereas it did increase with the foaming agent addition rate. It is considered that there may be an optimum addition rate. Furthermore, the optimum additive addition rate is expected to depend on the type of fly ash and thus requires further study.

### 3.4. Properties of Concrete with MFAS

The mechanical and physical properties of concrete with fly ash replacement ratios of 15% and 30% and different amounts of MFAS were measured.

**Figure 12:**
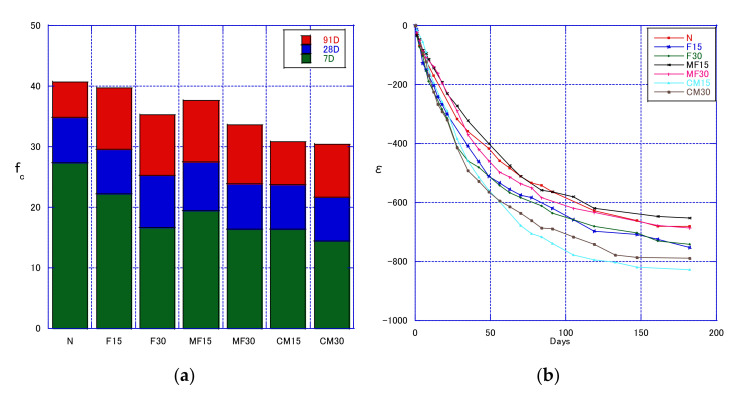
Compressive strength (**a**) and drying shrinkage (**b**) of concrete with modified fly ash slurry.

Figure 12a shows the compressive strength of concrete containing recycled aggregate and fly ash or modified fly ash at 7–91 days. Using fly ash reduced the compressive strength of concrete at 7–91 days, and a 30% replacement ratio decreased the compressive strength with more than a 15% replacement ratio. For a replacement ratio of 15%, although the strength for modified fly ash concrete was lower than that for normal fly ash concrete, the modified fly ash concrete strength increased faster. At 7, 28, and 91 days, the strengths of F15 (15% fly ash) were 81.1%, 84.7%, and 97.5%, whereas the strengths of M15 (15% modified fly ash concrete) were 71.2%, 79.1%, and 92.5%, respectively. For the replacement ratio of 30%, the growth rate of the strength of fly ash and modified fly ash concrete was the same. At 7, 28, and 91 days, the strengths of F30 (30% fly ash) were 60.7%, 72.5%, and 86.6%, whereas the strengths of M30 (30% modified fly ash concrete) were 60.0%, 68.5%, and 82.5%, respectively.

The use of MFA had little effect on the strength of concrete. This is because the chemical characteristics of fly ash have a large effect on pozzolanic reactivity [39], and as discussed above, the flotation does not change the chemical characteristics of FA. Adding 30% of MFA or FA had a greater impact on the strength of concrete, increasing it by more than 15%. However, for recycled concrete, the two different mixing amounts of MFA or FA showed a very minor difference in strength. In words, the impact on the strength of recycled concrete was very minor.

Figure 12b shows the drying shrinkage of concrete specimens containing recycled aggregate and fly ash or modified fly ash up to 118 days—see the F15 line and F30 line in the figure, which are both below the red line. Fly ash increased the drying shrinkage of concrete compared with the control. The distance between these two lines is small. At fly ash replacement ratios of 15–30%, the replacement ratio did not affect the drying shrinkage—see the C100M15 line and C100M30 line, which are both below the red line. The distance between these two lines is small. At fly ash replacement ratios of 15–30%, and a recycled aggregate replacement ratio of 100%, the fly ash replacement ratio did not affect the drying shrinkage. The distance between the two lines is small. This suggests that within a certain range (15–30%), for non-mixed aggregates, the influence of fly ash on drying shrinkage is constant. Therefore, the carbon content of FA is different, and the effect on the drying shrinkage of concrete is different. However, the amount of FA mixed does not have a huge impact on the drying shrinkage.

**Figure 13:**
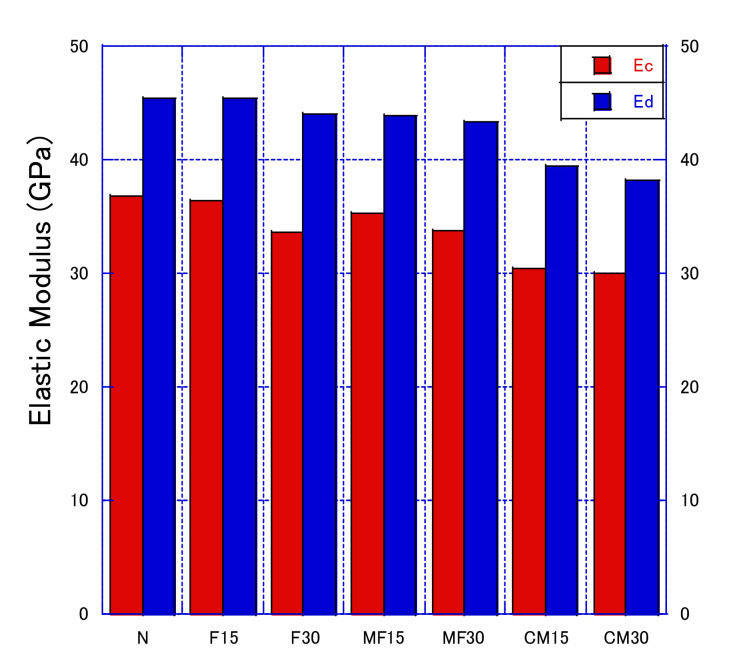
Static and Dynamic elastic Modulus.

Figure 13 shows the static elastic modulus and dynamic elastic modulus of concrete containing MFAS. The measurement of the static elastic modulus destroys the concrete test block, whereas the measurement of the dynamic elastic modulus does not; thus, measuring the dynamic elastic modulus is more convenient. Both measure the same characteristic of concrete, and thus showed consistent results. The effect of fly ash content on both moduli decreased as the fly ash content increased. The trend of the elastic modulus is consistent with the performance of the concrete’s compressive strength, indicating that there is a certain correlation between the static elastic modulus and the compressive strength, and the trend of the dynamic elastic modulus is also consistent, meaning that when predicting the compressive strength of concrete, the dynamic elasticity modulus can also be used as an important factor, and is not limited to ordinary concrete. For example, when Farooq et al. [40] predicted self-compacting concrete, the dynamic elastic modulus was considered as a factor.

## 4. Conclusions

We developed a prototype device for removing unburned carbon from fly ash by means of the flotation method and examined the operating conditions of the device experimentally. The fly ash was used in Portland cement concrete and the concrete properties were measured. Our findings are summarized as follows.

The base model, which used a circulating microbubble generator with a spiral pump, removed unburned carbon from fly ash by means of the froth flotation method without affecting the chemical composition of the fly ash. The removal efficiency was increased by adding a collecting agent to 60 wt% fly ash slurry and pre-stirring with a concrete mixer for 30 min. The LOI was greatly reduced within 10 min, and a treatment time of 30 min was sufficient. Flotation was improved by pre-stirring the sample and adding water to form a slurry with a concentration of 20 wt%.

MFAS was used in the mortar, and its properties were better than those of dry fly ash. MFAS reduced the compressive strength of concrete at 7–91 days. The drying shrinkage of concrete containing fly ash was greater than that of ordinary concrete; however, at fly ash replacement ratios of 15–30%, the replacement ratio did not affect the drying shrinkage. 

Our results demonstrate that it is feasible to use modified fly ash prepared using the flotation method in concrete.

## Figures and Tables

**Table 1:** Physical characteristics of fly ash.

Fly Ash	LOI (%)	Density (g/cm^3^)	Specific Surface Area (cm^2^/g)
a-1	0.72	2.30	1510
a-2	0.42	2.27	2970
a-3	1.30	2.29	2790
a-4	6.99	2.30	4640
a-5	3.86	2.24	4250
a-6	2.22	2.25	4200
b-a	5.87	2.12	4590
b-2	7.39	2.27	4890
b-3	7.85	2.37	4620
b-4	12.92	2.24	4890
c	7.25	2.3	5560
d	9.85	2.11	6060
e	2.81	2.11	3470
f	3.36	2.26	3280

**Table 2:** Properties of FA and MFA.

Type	MFA	Raw Ash
LOI (%)	1.75	13
Density (g/cm^3^)	2.33	2.32
Blaine (cm^2^/g)	3220	4830

**Table 3:** Mix proportion.

Type	W/C	W/B	Unit (kg/m^3^)						
	(%)	(%)	W	C	FA	MFA	S	G	RG
C0F0F0	55	55	180	327	-	0	857	945	-
C0F0F15	65	55		278	49		840	945	-
C0F0F30	79	55		229	98		824	945	-
C0F0M15	65	55		278	-	49	842	945	-
C0F0M30	79	55		229	-	98	828	945	-
C100F0M15	65	55		278	-	49	842	-	885
C100F0M30	79	55		229	-	98	828	-	885

**Table 4:** Physical characteristics of untreated ash and tail ash.

Type		LOI (%)	Density (g/cm^3^)	Specific Surface Area (cm^2^/g)
C-ash	Untreated ash	7.25	2.30	5560
	Tail ash	2.31	2.31	4660
D-ash	Untreated ash	9.85	2.11	6060
	Tail ash	2.88	2.20	4520

## Data Availability

The data presented in this study are available on request from the corresponding author. The data are not publicly available due to privacy restrictions.
